# Supplementary material for: Differential longitudinal establishment of human fecal bacterial communities in germ-free porcine and murine models
Source: Commun Biol. 2020 Dec 11;3:760. doi: 10.1038/s42003-020-01477-0 (PMC7733510; doi:10.1038/s42003-020-01477-0)
Supplement: Supplementary file 1 — Supplementary Information [file 42003_2020_1477_MOESM1_ESM.pdf]

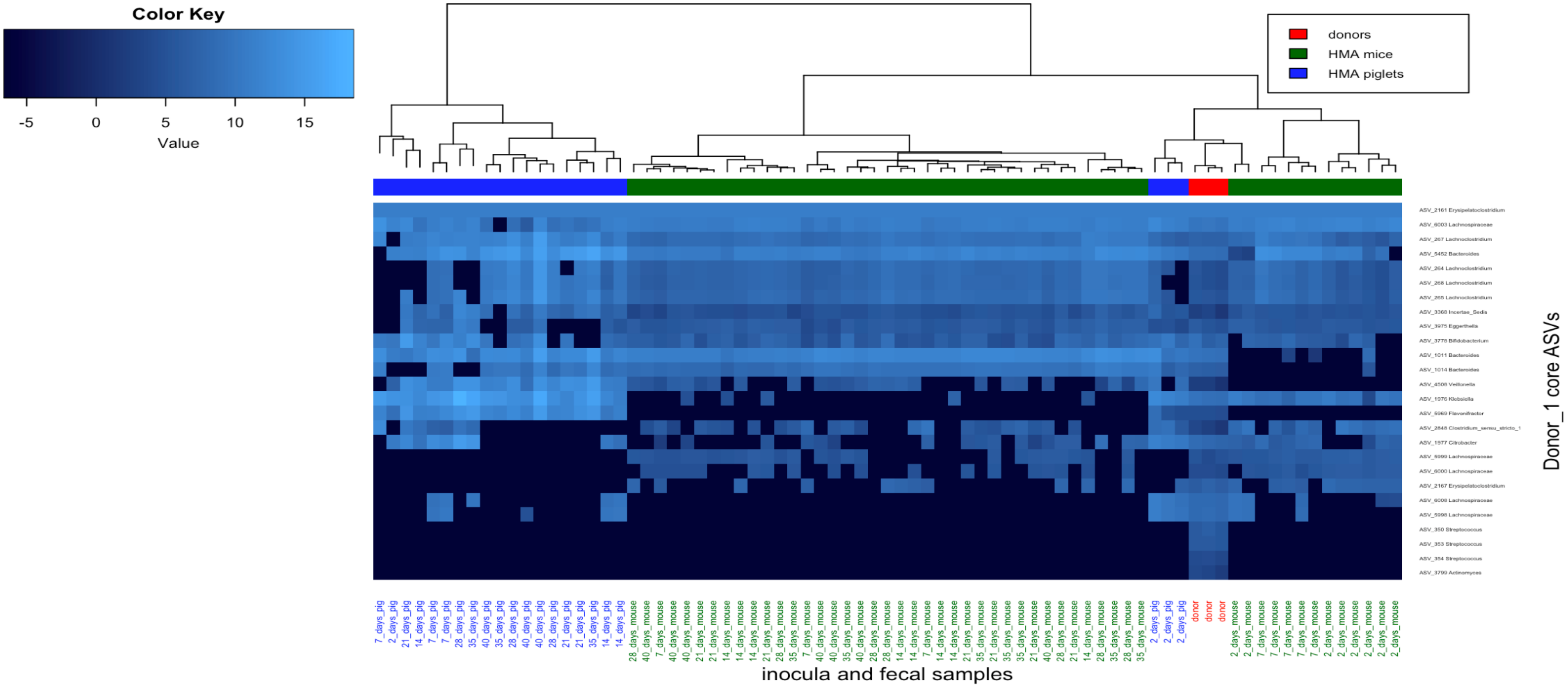

Supplementary Figure 1: Heatmap showing the distribution of core donor ASVs from Donor_1 across the corresponding HMA mouse and piglet fecal samples at different time points. The abundances have been normalized using variance-stabilizing transformation. The dendrogram depicts sample clustering based on Bray-Curtis distances.

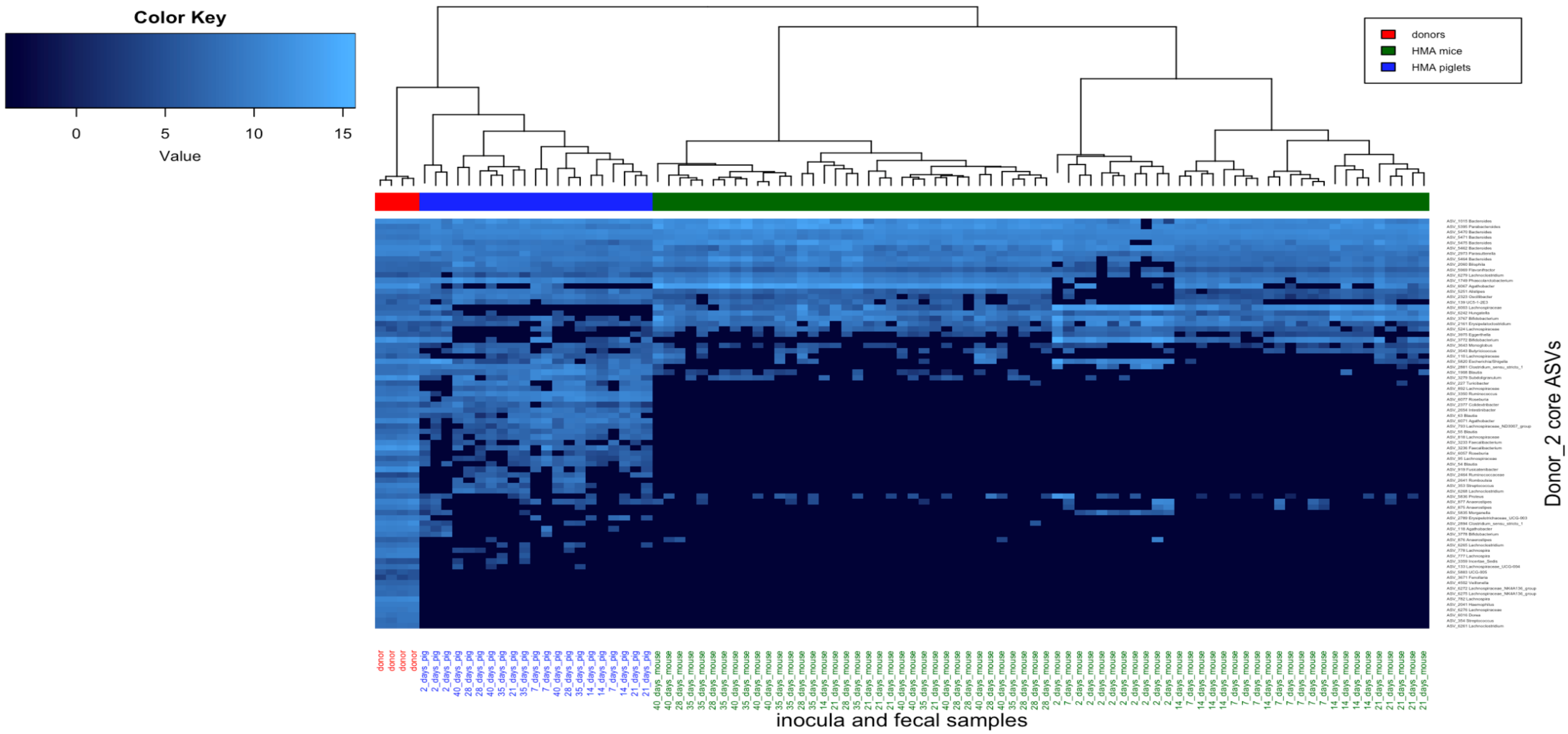

Supplementary Figure 2: Heatmap showing the distribution of core donor ASVs from Donor_2 across the corresponding HMA mouse and piglet fecal samples at different time points. The abundances have been normalized using variance-stabilizing transformation. The dendrogram depicts sample clustering based on Bray-Curtis distances.

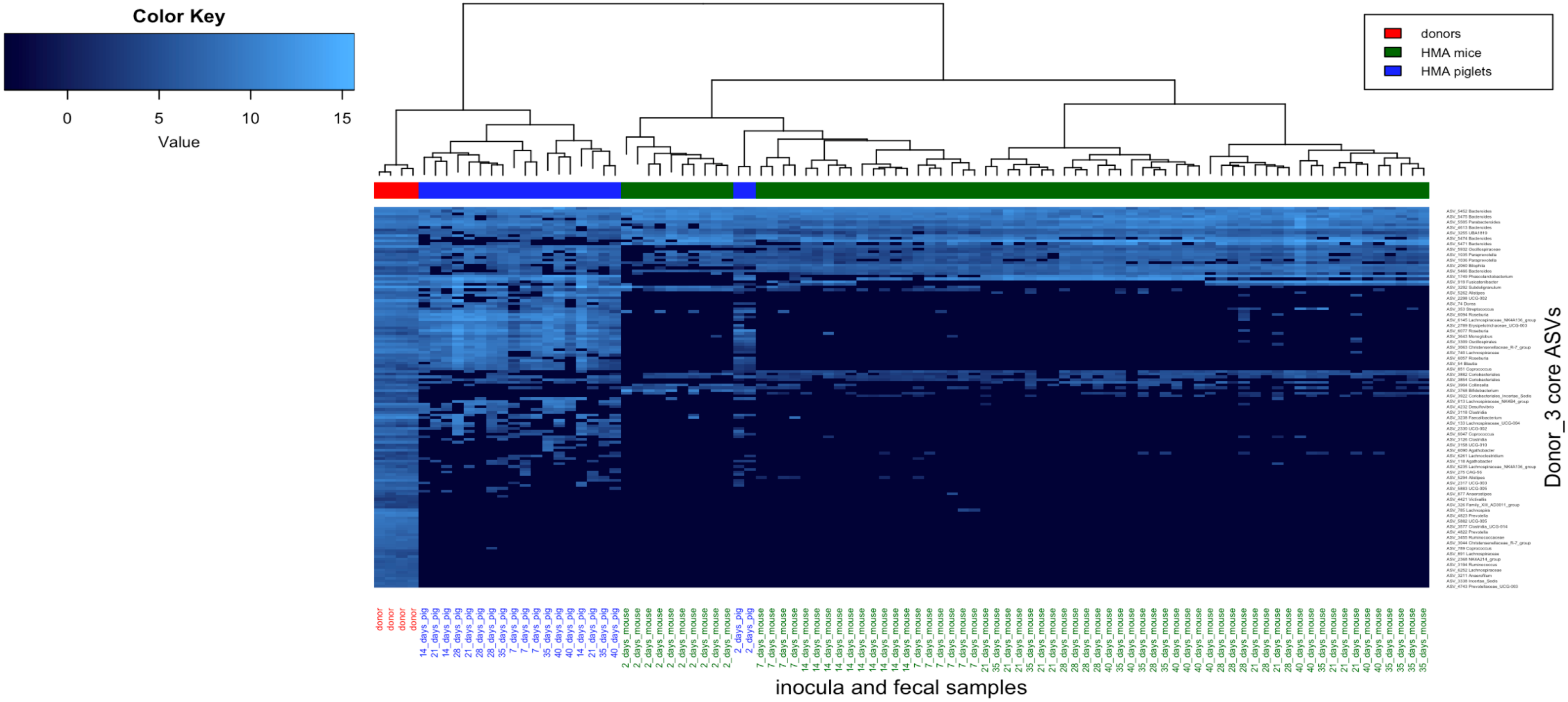

Supplementary Figure 3: Heatmap showing the distribution of core donor ASVs from Donor_3 across the corresponding HMA mouse and piglet fecal samples at different time points. The abundances have been normalized using variance-stabilizing transformation. The dendrogram depicts sample clustering based on Bray-Curtis distances.

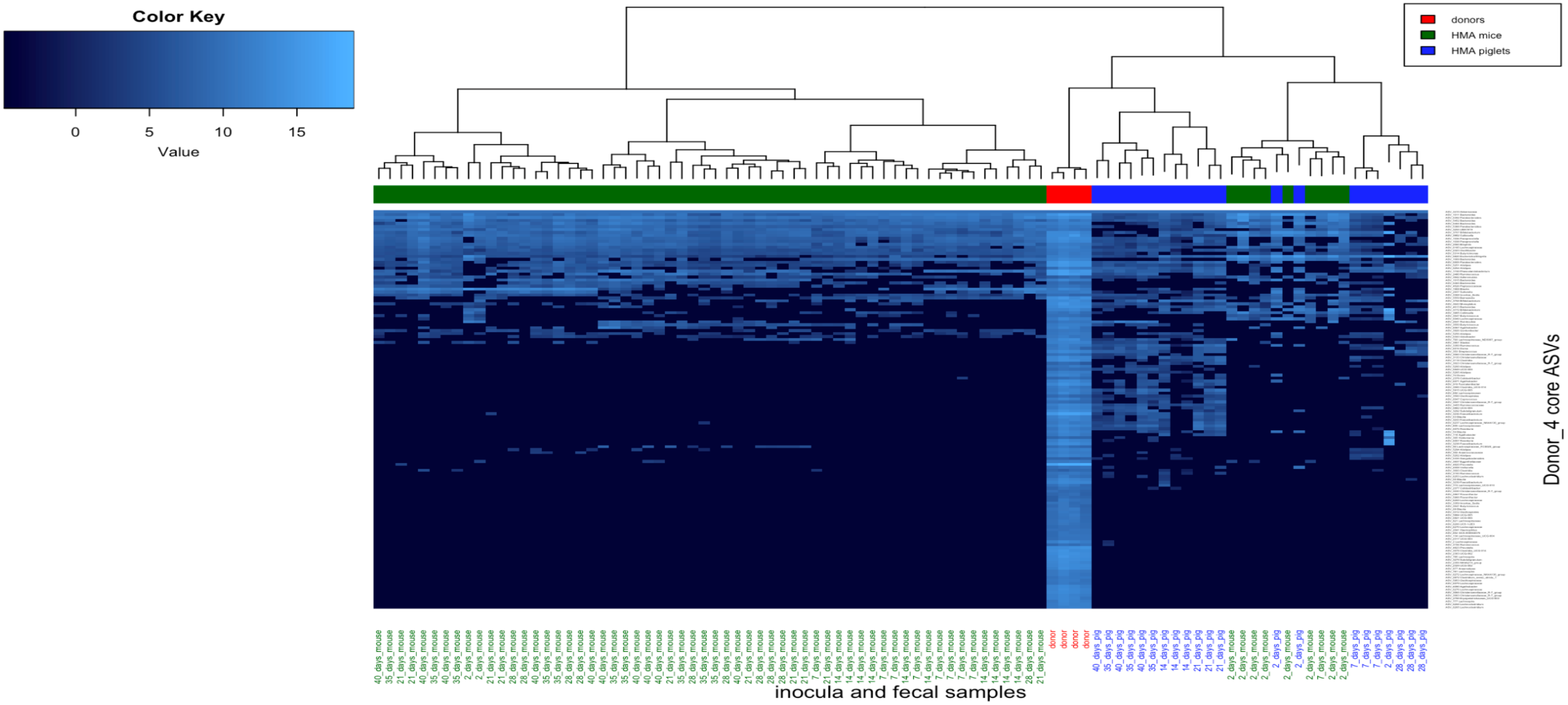

Supplementary Figure 4: Heatmap showing the distribution of core donor ASVs from Donor_4 across the corresponding HMA mouse and piglet fecal samples at different time points. The abundances have been normalized using variance-stabilizing transformation. The dendrogram depicts sample clustering based on Bray-Curtis distances.

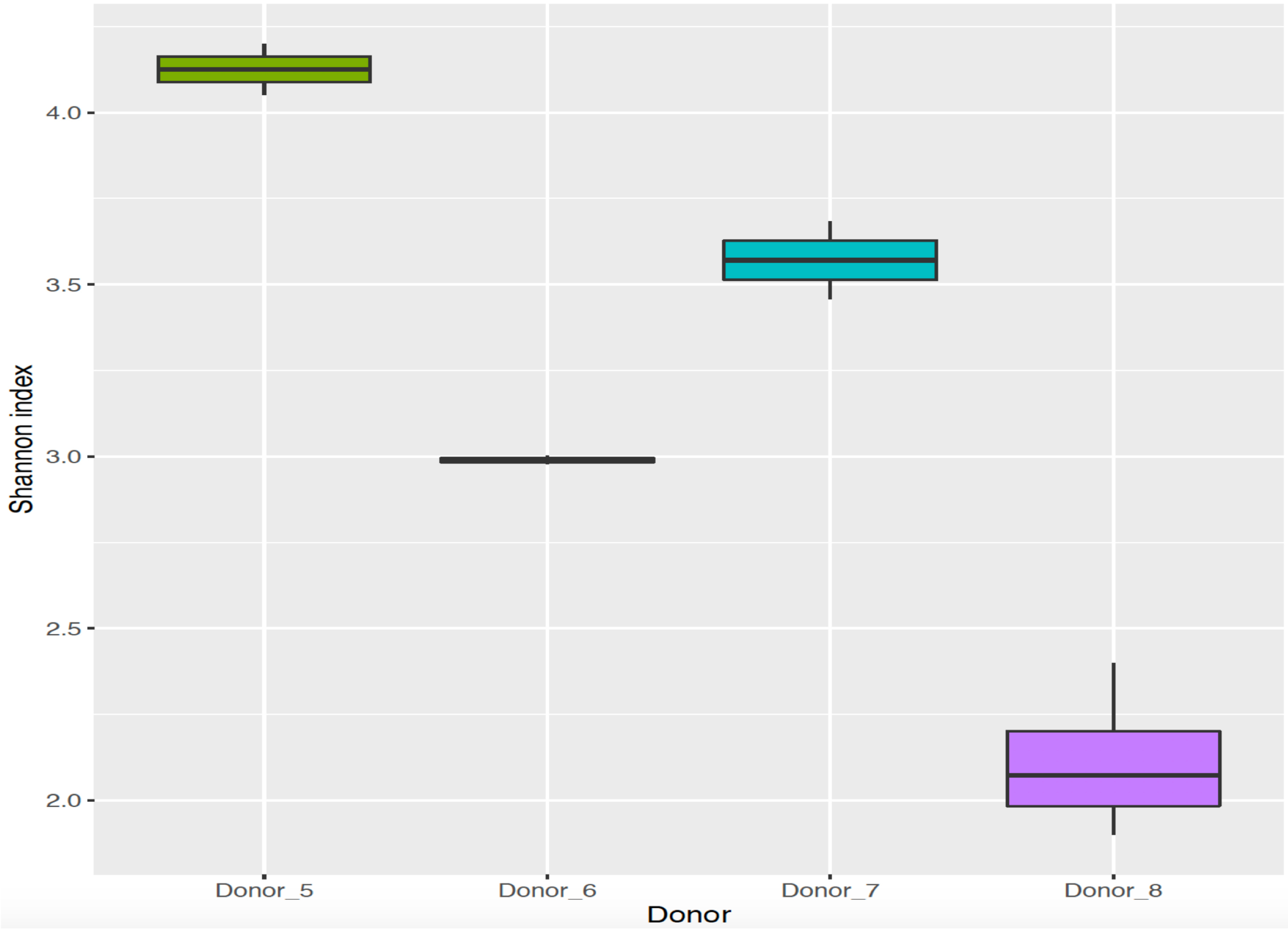

Supplementary Figure 5: Box-whisker plot comparing the alpha diversity of the inocula among the different donors of the second study using the Shannon index. Statistical comparisons were performed using the Wilcoxon rank-sum test. None of the donor diversities were statistically significantly different from each other. The box represents the 25th and 75th percentiles as the interquartile **r**ange (IQR) and the short black line represents the median. The whiskers represent the minimum and maximum values. Outliers are shown as black dots. n = 2 inoculum aliquots for Donor_5, Donor_6, and Donor_7; n = 4 inoculum aliquots for Donor_8.

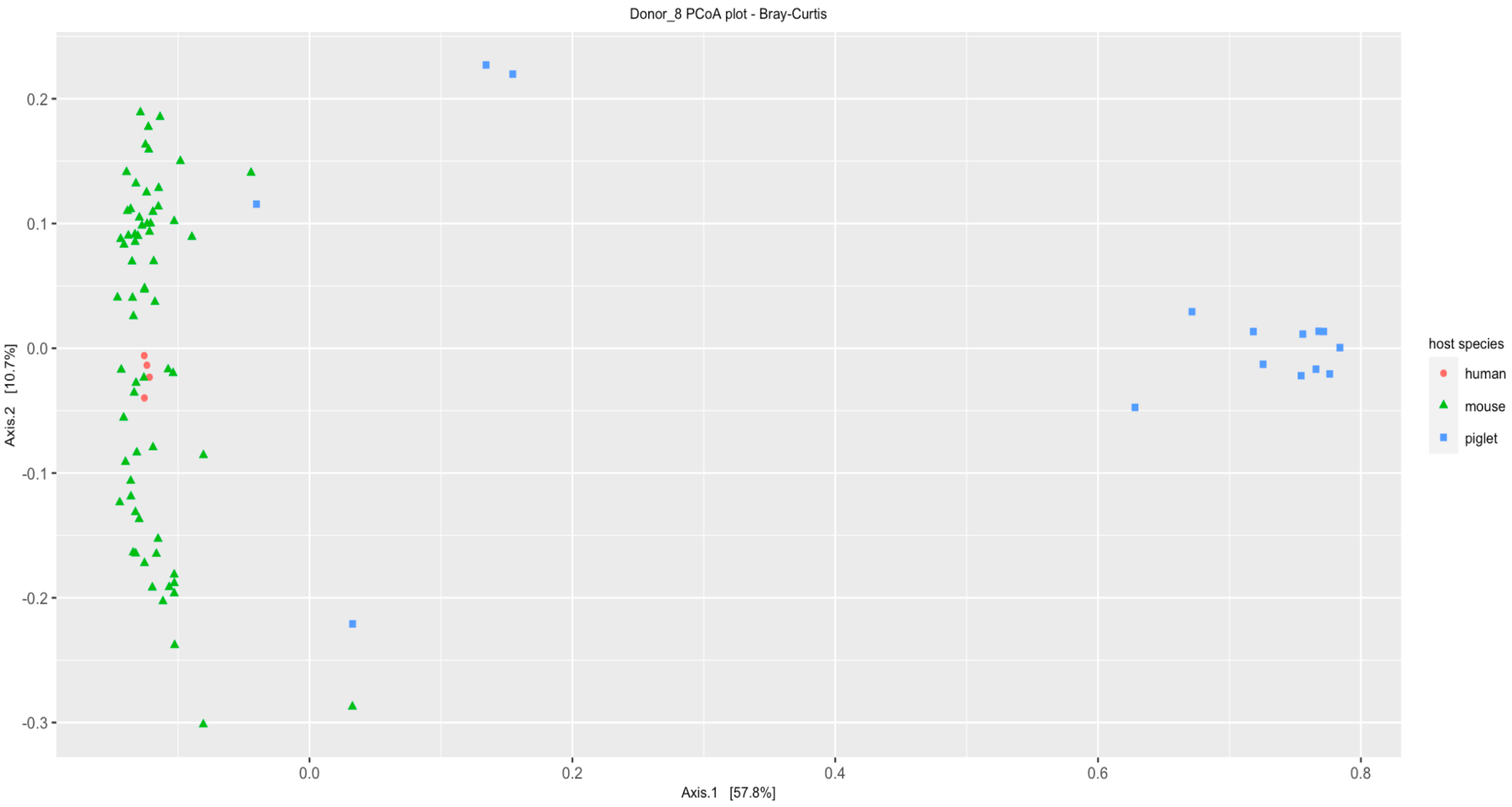

Supplementary Figure 6: Principal coordinate analysis plot based on Bray-Curtis distances comparing beta diversity of Donor_8 (infant) inocula with HMA mouse and HMA piglet fecal samples. *n* = 4 piglets and *n* = 10 mice. Red circles, human donor inocula; green triangles, HMA mouse fecal samples; blue squares, HMA piglet fecal samples.

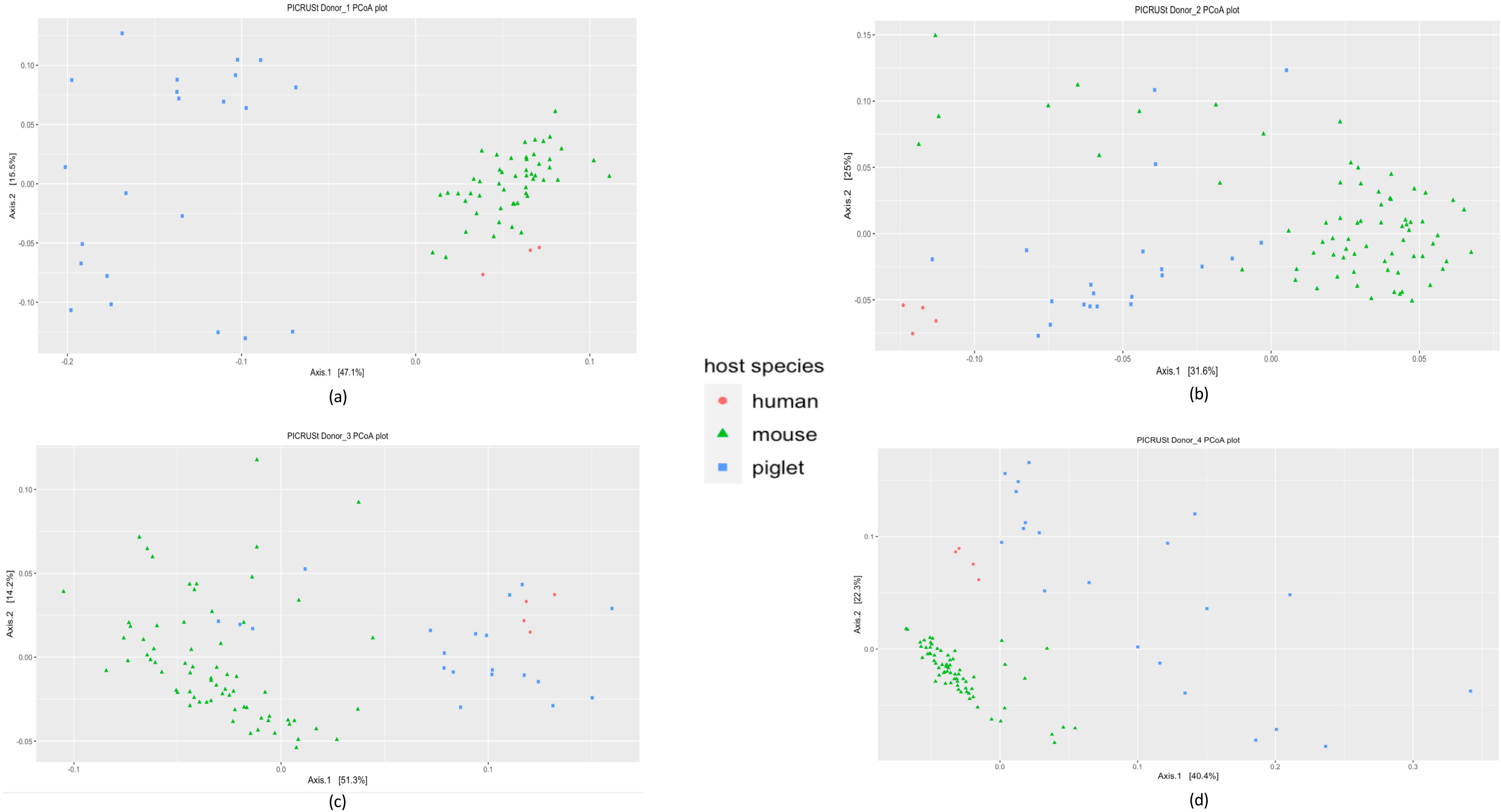

Supplementary Figure 7: Principal coordinate analysis (PCoA) plots based on Bray-Curtis distances comparing core PICRUSt-predicted KEGG orthology (KO) functions between the human donors and the HMA animal models. (a) Donor_1 (b) Donor_2 (c) Donor_3 and (d) Donor_4. n = 13 (3 piglets/donor, with the exception n=4 for Donor_1, 2 days post inoculation) and n= 37 mice (Donor_1=7, Donor_2 = 10, Donor_3 = 10, Donor_4 = 10). Red circles, human donor inocula; green triangles, HMA mouse fecal samples; blue squares, HMA piglet fecal samples.
