# Supplementary material for: Differential longitudinal establishment of human fecal bacterial communities in germ-free porcine and murine models
Source: Commun Biol. 2020 Dec 11;3:760. doi: 10.1038/s42003-020-01477-0 (PMC7733510; doi:10.1038/s42003-020-01477-0)
Supplement: Supplementary file 2 — Description of Additional Supplementary Files [file 42003_2020_1477_MOESM2_ESM.pdf]

Title: Supplementary Data 1 (.xlsx)

Description: Core ASV distribution for each donor in the two HMA animal models including the respective relative abundances and taxonomic classifications.

Title: Supplementary Data 2 (.xlsx).

Description: Phylum, family, and genus level groupings of colonizers and persistent colonizers in the two HMA animal models and their distribution in the human donors.

Title: Supplementary Data 3 (.xlsx).

Description: Mean relative abundances and taxonomic classifications for the persistent colonizers identified for each animal model within each donor.

Title: Supplementary Data 4 (.xlsx).

Description: Taxonomic classifications for core ASVs from each human donor that failed to colonize either of the two animal models.

Title: Supplementary Data 5 (.xlsx).

Description: Taxonomic classifications for 27 common core ASVs found across the 4 donors.

Title: Supplementary Data 6 (.xlsx).

Description: Colonization success of the 27 common core ASVs among the two HMA animal models.

Title: Supplementary Data 7 (.xlsx).

Description: Phylum, family, and genus level taxonomy assignments and relative abundances for the core ASVs from each donor that established in each animal model.

Title: Supplementary Data 8 (.xlsx).

Description: Relative abundance comparisons at each sampling time point for the persistent colonizers of each animal model for each donor.

Title: Supplementary Data 9 (.xlsx).

Description: Taxonomic classifications of persistent colonizers with donor-like abundances for each animal model.

Title: Supplementary Data 10 (.xlsx).

Description: Phylum, family, genus level taxonomy assignments for core ASVs from donors of subsequent study which colonized the two HMA animal models.

Title: Supplementary Data 11 (.xlsx).

Description: Diet composition.

Title: Supplementary Data 12 (.xlsx).

Description: Source data for main figures.
