# Supplementary material for: Differential longitudinal establishment of human fecal bacterial communities in germ-free porcine and murine models
Source: Commun Biol. 2020 Dec 11;3:760. doi: 10.1038/s42003-020-01477-0 (PMC7733510; doi:10.1038/s42003-020-01477-0)
Supplement: Supplementary file 15 — Reporting Summary [file 42003_2020_1477_MOESM15_ESM.pdf]

nature research

Corresponding author(s): Samodha C. Fernando

Last updated by author(s): Sep 30, 2020

Reporting Summary

Nature Research wishes to improve the reproducibility of the work that we publish. This form provides structure for consistency and transparency in reporting. For further information on Nature Research policies, seeAuthors & Referees and theEditorial Policy Checklist .

Statistics

For all statistical analyses, confirm that the following items are present in the figure legend, table legend, main text, or Methods section.

n/a	Confirmed	
☐	☒	The exact sample size (*n*) for each experimental group/condition, given as a discrete number and unit of measurement
☐	☒	A statement on whether measurements were taken from distinct samples or whether the same sample was measured repeatedly
☐	☒	The statistical test(s) used AND whether they are one- or two-sided<br>*Only common tests should be described solely by name; describe more complex techniques in the Methods section.*
☐	☒	A description of all covariates tested
☐	☒	A description of any assumptions or corrections, such as tests of normality and adjustment for multiple comparisons
☐	☒	A full description of the statistical parameters including central tendency (e.g. means) or other basic estimates (e.g. regression coefficient) AND variation (e.g. standard deviation) or associated estimates of uncertainty (e.g. confidence intervals)
☐	☒	For null hypothesis testing, the test statistic (e.g. *F*, *t*, *r*) with confidence intervals, effect sizes, degrees of freedom and *P* value noted<br>*Give P values as exact values whenever suitable.*
☒	☐	For Bayesian analysis, information on the choice of priors and Markov chain Monte Carlo settings
☒	☐	For hierarchical and complex designs, identification of the appropriate level for tests and full reporting of outcomes
☒	☐	Estimates of effect sizes (e.g. Cohen's *d*, Pearson's *r*), indicating how they were calculated

Our web collection on statistics for biologists contains articles on many of the points above.

Software and code

Policy information about availability of computer code

Data collection	No software was used.
Data analysis	All data analysis pipelines and codes are available as a R markdown file in the GitHub page of the Fernando Lab (https://github.com/FernandoLab/men_mice_and_pigs) along with all the metadata and accessory files.

For manuscripts utilizing custom algorithms or software that are central to the research but not yet described in published literature, software must be made available to editors/reviewers. We strongly encourage code deposition in a community repository (e.g. GitHub). See the Nature Research guidelines for submitting code & software for further information.

Data

Policy information about availability of data

All manuscripts must include a data availability statement. This statement should provide the following information, where applicable:
- Accession codes, unique identifiers, or web links for publicly available datasets
- A list of figures that have associated raw data
- A description of any restrictions on data availability

The datasets generated and analyzed during the current study are available under BioProject accession number PRJNA560867 from the National Center for Biotechnology Information (NCBI) sequence read archive (SRA).

Field-specific reporting

Please select the one below that is the best fit for your research. If you are not sure, read the appropriate sections before making your selection.

Life sciences study design

All studies must disclose on these points even when the disclosure is negative.

Sample size	No sample size determination was done prior to start of the experiment. The number of animals that can be maintained within gnotobiotic isolators is limited by logistical and animal welfare considerations. This was especially the case with the HMA piglets. However, the sample sizes (7-10 mice/treatment and 3-4 piglets/treatment) are comparable to what has been reported in the literature for similar studies.
Data exclusions	Three Donor_1-HMA mouse fecal samples were excluded from the data analysis due to low sequence read depth (<10,500 reads). Although the exact exclusion cutoff of <10,500 reads had not been established prior to commencement of the study, exclusion of samples with low sequencing depth after investigating the sequencing profiles had been planned.
Replication	Replication was performed to investigate the reproducibility of the percentages of 'colonizers' and 'persistent colonizers' for HMA mice by performing a second experiment with a different set of donors of similar age to the first set of donors. The findings were comparable with the exception of Donor_3 where the mice in the second experiment had much higher percentages of both 'colonizers' and 'persistent colonizers'. In addition, the beta diversity clustering patterns for the infant Donor_1 were successfully replicated with a second infant donor (Donor_8) and different groups of HMA mice and HMA piglets.
Randomization	In all experiments, both the GF mice and the GF piglets were randomly allocated to their specific treatment groups.
Blinding	Blinding was not relevant to this study as no comparisons were made between a 'Treatment' group and a 'control/placebo' group.

Reporting for specific materials, systems and methods

We require information from authors about some types of materials, experimental systems and methods used in many studies. Here, indicate whether each material, system or method listed is relevant to your study. If you are not sure if a list item applies to your research, read the appropriate section before selecting a response.

Materials & experimental systems

n/a	Involved in the study	
☒	☐	Antibodies
☒	☐	Eukaryotic cell lines
☒	☐	Palaeontology
☐	☒	Animals and other organisms
☐	☒	Human research participants
☒	☐	Clinical data

Methods

n/a	Involved in the study	
☒	☐	ChIP-seq
☒	☐	Flow cytometry
☒	☐	MRI-based neuroimaging

Animals and other organisms

Policy information about studies involving animals; ARRIVE guidelines recommended for reporting animal research

Laboratory animals	Male and female domestic piglets (Sus scrofa domesticus) and male and female mice (Mus musculus) of the C3H/HeN genetic background were used in this study.
Wild animals	This study did not involve any wild animals
Field-collected samples	Both HMA mice and HMA piglets were housed in flexible film isolators maintained under gnotobiotic conditions at the University of Nebraska-Lincoln (UNL) gnotobiotic mouse and pig facilities. Both groups of animals were provided the same irradiated solid diet (Teklad 2019 rodent diet) and autoclaved water ad libitum. A 14hr light/10hr dark cycle was provided for both the piglets and mice. The HMA piglets were initially maintained at 35 C for the first week after birth and then the temperature was reduced gradually to reach 25 C which was then maintained for the remainder of the study. The HMA mice were maintained at 20 - 24 C. Carbon dioxide inhalation was used as the euthanasia agent for the mice at the end of the study while injection of sodium pentobarbital was used to euthanize the HMA piglets.
Ethics oversight	All animal-related intervention protocols and husbandry practices were approved by the Institutional Animal Care and Use Committee (IACUC) at the University of Nebraska-Lincoln (protocols 1215, 1700 for mice; protocol 1400 for piglets).

Note that full information on the approval of the study protocol must also be provided in the manuscript.

Human research participants

Policy information about studies involving human research participants

Population characteristics	Human fecal donors were selected based on their age and on the basis of not having used antibiotics in the previous six months. Donors involved both male and female donors.

Recruitment	Participants self-selected/volunteered based on fulfillment of the age and antibiotic usage criteria mentioned above. Since the same donors were used to inoculate both groups of animals, no bias in self-selection on the part of the donors were expected to influence the results.
Ethics oversight	Human donors were recruited and consent was given as described on IRB protocol approval number 20170116853EP of the Institutional Review Board of the University of Nebraska-Lincoln.

Note that full information on the approval of the study protocol must also be provided in the manuscript.
